# Factors affecting uptake of optimal doses of sulphadoxine-pyrimethamine for intermittent preventive treatment of malaria in pregnancy in six districts of Tanzania

**DOI:** 10.1186/1475-2875-13-22

**Published:** 2014-01-14

**Authors:** Amon Exavery, Godfrey Mbaruku, Selemani Mbuyita, Ahmed Makemba, Iddajovana P Kinyonge, Hadija Kweka

**Affiliations:** 1 Ifakara Health Institute (IHI), Plot 463, Kiko Avenue, off Mwai Kibaki Road, Mikocheni, PO Box 78373, Dar es Salaam, Tanzania

**Keywords:** Malaria, Pregnancy, IPTp, SP, Tanzania

## Abstract

**Background:**

Intermittent preventive treatment during pregnancy (IPTp) with optimal doses (two+) of sulphadoxine-pyrimethamine (SP) protects pregnant women from malaria-related adverse outcomes. This study assesses the extent and predictors of uptake of optimal doses of IPTp-SP in six districts of Tanzania.

**Methods:**

The data come from a cross-sectional survey of random households conducted in six districts in Tanzania in 2012. A total of 1,267 women, with children aged less than two years and who had sought antenatal care (ANC) at least once during pregnancy, were selected for the current analysis. Data analysis involved the use of Chi-Square (χ^2^) for associations and multivariate analysis was performed using multinomial logistic regression.

**Results:**

Overall, 43.6% and 28.5% of the women received optimal (two+) and partial (one) doses of IPTp-SP respectively during pregnancy. Having had been counseled on the dangers of malaria during pregnancy was the most pervasive determinant of both optimal (RRR = 6.47, 95% CI 4.66-8.97) and partial (RRR = 4.24, 95% CI 3.00-6.00) uptake of IPTp-SP doses. Early ANC initiation was associated with a higher likelihood of uptake of optimal doses of IPTp-SP (RRR = 2.05, 95% CI 1.18-3.57). Also, women with secondary or higher education were almost twice as likely as those who had never been to school to have received optimal SP doses during pregnancy (RRR = 1.93, 95% CI 1.04-3.56). Being married was associated with a 60% decline in the partial uptake of IPTp-SP (RRR = 0.40, 95% CI 0.17-0.96). Inter-district variations in the uptake of both optimal and partial IPTp-SP doses existed (P < 0.05).

**Conclusion:**

Counseling to pregnant women on the dangers of malaria in pregnancy and formal education beyond primary school is important to enhance uptake of optimal doses of SP for malaria control in pregnancy in Tanzania. ANC initiation in the first trimester should be promoted to enhance coverage of optimal doses of IPTp-SP. Programmes should aim to curb geographical barriers due to place of residence to enhance optimal coverage of IPTp-SP in Tanzania.

## Background

Malaria in pregnancy (MiP) exerts major public health concerns worldwide, particularly in malaria-endemic settings, and results in adverse health outcomes for both woman and foetus [1,2]. Low birth weight (LBW), infant mortality, maternal anaemia, spontaneous abortion, and stillbirth are documented as devastating health consequences of *Plasmodium falciparum* MiP [3]. About 11% of neonatal mortality in malaria-endemic African countries is attributable to LBW that results from MiP [4]. In sub-Saharan Africa, MiP reportedly accounts for 26% of severe maternal anaemia [2] and up to 200,000 infant mortality annually due to LBW [5,6]. Some 10,000 maternal deaths are recorded each year due to malaria-related anaemia [5]. Against this, research has shown that intermittent preventive treatment during pregnancy (IPTp) with two doses of sulphadoxine-pyrimethamine (SP) protects pregnant women from maternal anaemia and malaria placental infection, and also reduces the incidence of LBW [6]. Current evidence reveals further that exposure to IPTp with SP (IPTp-SP) and insecticide-treated bed nets (ITNs) are associated with reductions in both neonatal mortality and LBW [7]. Under trial conditions, IPTp-SP is acknowledged to be effective in reducing neonatal mortality [8]. Recent evidence from Malawi reveals that IPTp-SP enhances birth outcomes [9].

The World Health Organization (WHO) recommends IPTp-SP for all pregnant women at each schedule of antenatal care (ANC), with the first dose administered as early as possible during the second trimester of gestation, and that the subsequent dose be given at least one month after the first. It is clarified further that the last dose can be administered up to the time of delivery, with no safety concerns [10]. SP is used for IPTp in many sub-Saharan African countries [11]. In Tanzania where neonatal mortality remains one of the challenging public health concerns [3,12], the national policy stipulates provision of SP to all pregnant women during ANC visits [13], between 20 and 24 weeks’ gestation for the first dose and between 28 and 32 weeks’ gestation for the second dose to properly prevent malaria [14].

Several factors associated with incomplete coverage of IPTp have been reported in previous studies. These include late ANC initiation [15,16], multigravidity [17] and lack of knowledge of adverse consequences of MiP [18]. Systemic factors, namely, workforce shortage, inadequate or erratic drug supply, poor skills of providers, and skewed access to ANC services [15] have been identified as well. One publication based on a nationally representative household survey in Tanzania reported a decline between 2005 and 2007 for both IPTp-1 and IPTp-2: 71% versus 65% and 38% versus 30% respectively [19]. The study found that urban residence was the only individual factor associated with IPTp-2. The study however made its inferences based on Chi-Square (χ^2^) tests, without involving multivariate regression analysis to take care of confounding. Another study estimated coverage of IPTp-1 and IPTp-2 at 70 and 35%, respectively, in 2005/06 [20], but did not identify factors affecting each. Generally, research documentation of factors influencing both partial and optimal use of SP for malaria control in pregnancy is limited, and even more so for the latter. It is further unknown whether correlates for partial, and optimal use of SP are similar or different.

Although the majority of women attend antenatal clinic for ANC at least once during pregnancy, extant evidence shows that IPTp-SP uptake as well as ITN coverage among pregnant women is unacceptably low in most countries [21], and lowest in areas with highest transmission of malaria [22]. The Roll Back Malaria (RBM) Summit, which was held in Abuja, Nigeria in 2000, targeted 60% coverage of recommended MiP prevention and treatment by 2005 [23,24], a target which was later set to 80% by 2010 [25]. Although almost all pregnant women in Tanzania receive ANC at least once and 43% at least four times [3], 66% of the women in Tanzania mainland took anti-malarial drugs at least one dose during pregnancy, and 27% took the recommended (two or more) dosage of SP in 2010 [3]. This reflects a major missed opportunity that warrants analysis of factors that influence uptake, particularly optimal uptake of IPTp-SP, in order to inform programmes and policies of underlying contexts that should be observed to ensure universal and optimal coverage of IPTp-SP. Also earlier studies have made their inferences based on descriptive analyses with limited use of such sophisticated methods of data analysis as regressions. This study seeks to: (1) estimate the extent of IPTp-SP uptake as a proportion of women that received one dose, and two or more (two+) doses of SP during pregnancy; and, (2) conduct multivariate analysis using multinomial logit model (polytomous logistic regression) to identify factors affecting partial and optimal use of IPTp-SP among pregnant women in six districts (Geita, Kahama, Kondoa, Mbozi, Singida and Sumbawanga) of Tanzania.

## Methods

### Data and area

The data for this study were collected in 2012 in six Tanzanian rural districts namely Geita, Kahama, Kondoa, Mbozi, Singida and Sumbawanga. The geographical locations of the districts, which are all malaria endemic, represent three of eight health zones as described by the Tanzania Ministry of Health and Social Welfare. The data were collected as part of a large cross-sectional household survey to serve baseline purposes for the Empower II Project implemented by the Ifakara Health Institute, in Tanzania. The project seeks to improve maternal, newborn and child health (MNCH) services for women of reproductive age and children aged less than five years in the stud area. It implements MNCH proven interventions to demonstrate how best such interventions can be scaled up across the country. Under the maternal component it helps to build capacity of district health managers to effectively deliver maternal health services in the continuum of care including malaria during pregnancy. Data collection tools were quantitative and there were field interviewers to interview each of the respondents sampled. Sampling for the survey was random and was implemented using probability proportionate to size (PPS). This method is used when sampling units (e.g. districts, villages, etc.) are of different sizes in order to ensure that the resulting sample is representative of each unit. Data entry was done in Microsoft Office Access and latter transferred in STATA statistical software for cleaning and analysis.

### Population

Women who had children below the age of two years at the time of the survey and had sought ANC at least once during pregnancy were selected from the main database for the current analysis. Having sought ANC at least once was considered an important criterion because questions pertaining to MiP and IPTp were administered to women who did so.

### Variables

The dependent or outcome variable for this study was uptake of IPTp-SP. This was defined as the extent of SP utilization for malaria control during pregnancy and was derived from the question

“*During pregnancy of (NAME OF CHILD), did you use drugs (SP) to prevent malaria?*”

If yes, “*how many times*?” Responses were grouped in three categories such that:

$$\mathrm{Uptake}\;\mathrm{of}\;\mathrm{IPTp}-\mathrm{SP}=\left\{\begin{array}{l} \;\mathrm{NONE}\;\mathrm{if}\;\mathrm{no}\;\mathrm{SP}\;\mathrm{dose}\;\mathrm{was}\\ \;\mathrm{taken}\;\mathrm{during}\;\mathrm{pregnancy}\\ \;\mathrm{PARTIAL}\;\mathrm{if}\;\mathrm{only}\;\mathrm{one}\;\mathrm{dose}\\ \;\mathrm{of}\;\mathrm{SP}\;\mathrm{was}\;\mathrm{taken}\;\mathrm{during}\\ \;\;\mathrm{pregnancy}\\ \;\mathrm{OPTIMAL}\;\mathrm{if}\;\mathrm{two}\;\mathrm{or}\;\mathrm{more}\;\\ \;\left(2+\right)\;\mathrm{doses}\;\mathrm{of}\;\mathrm{SP}\;\mathrm{were}\;\mathrm{taken} \end{array}\right)$$

Independent variables included maternal age, marital status, maternal education, maternal occupation and district of residence. Others were whether a woman was counseled on the dangers of malaria during pregnancy, number of ANC visits made, timing of ANC initiation, and pregnancy intentions.

### Statistical analysis

The data were analysed both descriptively and analytically using standard methods of applied statistics in public health. Frequency distribution (one-way tabulations) of participants across background characteristics was performed first, then bivariate analysis was conducted, in which the outcome variable, IPTp-SP uptake, was cross-tabulated against each of the independent variables. The degree of association between each pair of variables was tested using Pearson’s Chi-Square (χ^2^) because all variables were categorical. In this process, the degree of IPTp-SP uptake was compared across categories of each of the independent variables. Where the test of association between the outcome and each of the independent variables showed a P-value of 5% or less, the null hypothesis of no association between the variables was rejected and consequently concluded that they were significantly associated, otherwise no association was deduced.

The data were finally subjected to regression analysis using multinomial logistic regresion in a multivariable way. This was to ensure that variables were adjusted for one another to obtain independent predictors of the IPTp-SP uptake. The category ‘none’ of the outcome variable was made a baseline/pivot outcome thus assessing what predicts partial, and complete receipt of IPTp-SP doses. Selection of independent variables for the multivariate models relied on each one’s ability to improve the overall model. This was achieved through the use of log-likelihood ratio test. From the model outputs, relative risk ratios (RRR), their corresponding 95% confidence intervals (CI) and P-values were all presented. Significance level was set at 5%. The whole process of data analysis was conducted using STATA (version 11) statistical software.

### Ethical consideration

The primary study was approved by the Medical Research Coordinating Committee (MRCC) of the National Institute for Medical Research (NIMR) in Tanzania. During data collection, participation was voluntary, with potential respondents having to sign an informed consent form first. The interviewer read, and explained the content of the consent form to the potential respondent. The respondent was also free to read the consent form by herself and ask for clarifications concerning any aspect of the content. Then an interview followed only if the respondent agreed and signed the consent form to take part in the survey. The data were managed carefully and remained anonymous throughout.

## Results

### Background characteristics

A total of 1,267 women who had children aged less than two years and had sought ANC at least once during pregnancy were analysed for the current question. Mean age of the participants was 27.2 ±7.4 years, ranging from 15 to 50. The majority of women (90%) were married and about 70% of the women had a primary education. Occupationally, a majority of the women (72%) were farmers or livestock keepers. With respect to district of residence, 15.6% of the women resided in Geita, 29.0% resided in Kahama, 17.1% resided in Kondoa, 22.1% resided in Mbozi, 6.5% resided in Singida and 9.6% resided in Sumbawanga (Table 1).

**Table 1:** Profile of respondents

	**Number of respondents (n)**	**Percent (%)**
**Overall**	**1,267**	**100.0**
**Age (years)**		
<20	132	10.4
20-34	909	71.7
>34	226	17.8
**Marital status**		
Currently married	1,152	90.9
Ever married	50	4.0
Single	65	5.1
**Education**		
No education	226	17.8
Primary	879	69.4
Secondary+	162	12.8
**Occupation**		
Farmer/livestock keeper	909	71.7
Employed/self-employed	165	13.0
No job/housewife	193	15.2
**District**		
Geita	198	15.6
Kahama	368	29.0
Kondoa	217	17.1
Mbozi	280	22.1
Singida	82	6.5
Sumbawanga	122	9.6

### Bivariate analysis

Table 2 displays percentage distribution of IPTp-SP uptake by characteristics of respondents. Overall, 43.6 and 28.5% of the women received complete and partial doses of SP, respectively, during pregnancy. This was equivalent to 72.1% coverage of IPTp-SP of at least one dose. These proportions were significantly influenced by various characteristics. Receipt of both partial and optimal IPTp-SP doses was highest among women who were counseled on the dangers of MiP in comparison of those who were not counseled, 31.7% versus 20.7% and 51.7% versus 23.7% respectively (P <0.001).

**Table 2:** Per cent distribution of IPTp-SP uptake by characteristics of respondents (n = 1,267)

**Characteristic**	**Number of respondents**	**IPTp-SP uptake**			**P-value**
		**None**	**Partial (1 dose)**	**Optimal (2+ doses)**	
**OVERALL**	**1,267**	**27.9**	**28.5**	**43.6**	**-**
**Counseled on dangers of MiP**					
No	367	55.6	20.7	23.7	<0.001
Yes	900	16.7	31.7	51.7	
**Timing of ANC initiation**					
First trimester	222	20.3	33.3	46.4	0.015
Second trimester	856	29.0	26.6	44.4	
Third trimester	189	32.3	31.2	36.5	
**Number of ANC visits made**					
1-3	666	28.5	31.5	39.9	0.011
4+	601	27.3	25.1	47.6	
**Pregnancy intention**					
Unintended/don’t remember	368	31.3	26.4	42.4	0.220
Intended	899	26.6	29.4	44.1	
**Age (years)**					
<20	132	33.3	24.2	42.4	0.494
20-34	909	27.4	28.4	44.2	
>34	226	27.0	31.4	41.6	
**Marital status**					
Currently married	1,152	27.2	29.5	43.3	0.015
Ever married	50	46.0	16.0	38.0	
Single	65	27.7	20.0	52.3	
**Education**					
No education	226	38.5	22.6	38.9	<0.001
Primary	879	27.4	30.4	42.2	
Secondary+	162	16.1	26.5	57.4	
**Occupation**					
Farmer/livestock keeper	909	29.4	28.9	41.7	0.040
Employed/self-employed	165	18.8	28.5	52.7	
No job/housewife	193	29.0	26.4	44.6	
**District**					
Geita	198	46.5	18.7	34.9	<0.001
Kahama	368	18.2	27.5	54.4	
Kondoa	217	26.3	30.0	43.8	
Mbozi	280	22.1	39.6	38.2	
Singida	82	24.4	36.6	39.0	
Sumbawanga	122	45.9	13.9	40.2	

Timing of ANC initiation was significantly important in determining the extent of IPTp-SP uptake (P = 0.015). Uptake of optimal doses of IPTp-SP was highest among women who initiated ANC in the first trimester of pregnancy and lowest among those who initiated ANC in the third trimester (46.4% versus 36.5%). Similarly, women who had sought ANC at least four times in the course of pregnancy were more likely than those who received ANC less than four times to receive optimal doses of IPTp-SP (47.6 against 39.9%). In contrast, receipt of partial IPTp-SP doses was higher among women who received ANC less than four times than that observed among women who had sought ANC at least four times during pregnancy (31.5 against 25.1%) (P = 0.011).

In terms of marital status, receipt of complete IPTp-SP doses was highest among women who were single (52.3%), whereas partial IPTp-SP doses became lowest at 16.0% among those ever married (P = 0.015). With regard to education, uptake of IPTp-SP increased with education, from as low as 38.9% among those who had no education to as high as 52.3% among those with secondary and higher education. There was no clear pattern of uptake of partial IPTp-SP doses, but was highest at 30.4% among women that had primary education (P <0.001). Other factors associated with IPTp-SP uptake were occupation (P = 0.040) and district of residence (P <0.001), whereby partial uptake of IPTp-SP ranged from 13.9% in Sumbawanga to 39.6% in Mbozi, and optimal uptake of IPTp-SP ranged from 34.9% in Geita to 54.4% in Kahama district.

### Multivariate analysis

Table 3 presents findings from the multivariate multinomial (polytomous) logistic regression models of factors associated with complete and partial uptake of IPTp-SP in the study area. Having had been counseled on dangers of MiP was the most pervasive determinant of both complete and partial uptake of IPTp-SP. The relative risk of receiving complete IPTp-SP doses was significantly 6.47 times higher among women who were counseled on dangers of MiP than that observed among women who were not counseled (RRR = 6.47, 95% CI 4.66-8.97). Likewise, receipt of partial IPTp-SP doses was 4.24 times more likely among women counseled on dangers of MiP than women not counseled (RRR = 4.24, 95% CI 3.00-6.00). Receipt of complete IPTp-SP doses was two-fold more likely among women that initiated ANC in the first trimester compared to women that initiated ANC in the third trimester (RRR = 2.05, 95% CI 1.18-3.57).

**Table 3:** Multivariate multinomial logistic regression models of correlates of IPTp-SP uptake in six districts of Tanzania, (n = 1,267)

	**IPTp-SP uptake**			
**Variable**	**Partial *versus *** **none**		**Optimal *versus *** **none**	
	**Relative risk ratio (RRR)**	**95% Confidence interval (CI)**	**Relative risk ratio (RRR)**	**95% Confidence interval (CI)**
**Counseled on dangers of MiP**				
No (ref)	1.00	-	1.00	-
Yes	4.24***	3.00-6.00	6.47***	4.66-8.97
**Timing of ANC initiation**				
First trimester	1.66*	0.94-2.94	2.05**	1.18-3.57
Second trimester	1.03	0.66-1.59	1.50*	0.98-2.29
Third trimester (ref)	1.00	-	1.00	-
**Age (years)**				
<20 (ref)	1.00	-	1.00	-
20-34	1.40	0.82-2.40	1.34	0.83-2.17
>34	1.50	0.80-2.80	1.29	0.73-2.20
**Marital status**				
Currently married (ref)	1.00	-	1.00	-
Ever married	0.40**	0.17-0.96	0.60	0.30-1.22
Single	0.51*	0.23-1.11	0.83	0.42-1.60
**Education**				
No education (ref)	1.00	-	1.00	-
Primary	1.22	0.78-1.89	1.17	0.79-1.74
Secondary+	1.44	0.73-2.83	1.93**	1.04-3.56
**Occupation**				
Farmer/livestock keeper	1.00	-	1.00	-
Employed/self-employed	0.98	0.57-1.71	1.03	0.62-1.74
No job/housewife	0.79	0.49-1.26	0.83	0.54-1.28
**District**				
Geita (ref)	1.00	-	1.00	-
Kahama	3.26***	1.91-5.55	3.26***	2.03-5.23
Kondoa	2.11**	1.20-3.70	1.55*	0.93-2.58
Mbozi	2.63***	1.54-4.51	1.25	0.76-2.06
Singida	2.86**	1.39-5.91	1.62	0.80-3.25
Sumbawanga	0.66	0.32-1.33	1.00	0.57-1.74

***P<0.001, **P<0.050, *P<0.100; ref = reference/baseline category.

Marital status was a significant predictor of partial IPTp-SP uptake, such that women who were ever married (currently divorced or widowed) were 60% less likely than those currently married to receive partial IPTp-SP (RRR = 0.40, 95% CI 0.17-0.96).

Education was also a significant predictor of optimal but not partial IPTp-SP uptake. Women with secondary and higher education were almost twice as likely as those who had had never been to school for formal education to receive complete IPTp-SP doses (RRR = 1.93, 95% CI 1.04-3.56).

Regarding district of residence, its effect was mostly on partial uptake of IPTp-SP, whereby the likelihood of uptake of partial IPTp-SP doses was significantly higher in Kahama (RRR = 3.26, 95% CI 1.91-5.55), Kondoa (RRR = 2.11, 95% CI 1.20-3.70), Mbozi (RRR = 2.63, 95% CI 1.54-4.51), and Singida (RRR = 2.86, 95% CI 1.39-5.91) than in Geita. Sumbawanga had less chance for partial IPTp-SP uptake compared to Geita, but the effect was not statistically significant (P >0.05). The district effect on receipt of complete doses of IPTp-SP was 3.26 times higher in Kahama compared to Geita (RRR = 3.26, 95% CI 2.03-5.23). The rest of the districts had higher chances for complete IPTp-SP doses compared to Geita, but none of the effects was statistically significant (P >0.05).

## Discussion

This study assessed the extent and factors affecting the uptake of both partial and optimal IPTp-SP doses among women during pregnancy in Geita, Kahama, Kondoa, Mbozi, Singida and Sumbawanga districts of Tanzania. Overall 43.6% of the women in the study area received optimal (two+) IPTp-SP doses during pregnancy. The rest, 28.5 and 27.9%, received partial (only one dose) and none of the SP doses, respectively. Therefore IPTp-SP coverage of at least one dose stood at 72.1%. These findings were higher than the national estimates from the 2010 Tanzania DHS report [3], reflecting geographical variations in the coverage of IPTp-SP in Tanzania. The survey reported 66.0% coverage of at least one dose (IPTp-1) and 27.0% for the recommended dosage (IPTp-2) [3]. These findings reveal an outstanding failure to meet the RBM target of 80% coverage of the recommended IPTp-SP dosage by 2010 [25]. This is an important missed opportunity, especially for women who received only one dose of IPTp-SP, meriting the need to understand why some women that receive one dose do not take the second. The understanding of what it would take to ensure that pregnant women who take the first dose of SP also take the second, and non-users initiate and sustain the dosage is crucial.

Several factors associated with both partial and optimal use of SP for malaria control in pregnancy were identified. Having had been counseled on the dangers of MiP was the strongest factor determining both optimal and partial uptake of IPTp-SP in the study area. The effect of this variable on optimal IPTp-SP was much higher than that for partial IPTp-SP. Likewise, education beyond primary school (i.e. secondary or higher) showed a remarkable influence on the optimal use of SP for malaria control in pregnancy, but had no significant effect on IPTp-1. Unfortunately, no similar or dissimilar findings were found in the literature linking counseling on the dangers of MiP during pregnancy and uptake of SP for IPTp. Creating and promoting awareness of the women, and probably the general public, on the dangers of MiP can potentially improve the uptake of optimal doses of SP and ultimately enhance health outcomes. Improving health services to enable, among others, better counseling services to pregnant women on the dangers of MiP could attract more women to take optimal doses of SP for the control of MiP. This goes together with a need to support women’s formal education beyond primary school to ensure autonomy and emancipation against restrictive cultures or lack of information that directly or indirectly retards access to better health care. Education is known to play a transformational role and stands as a catalyst for change that informs and influences decisions and choices [26,27].

This study observed that ANC initiation in the first trimester (proper timing) was significantly associated with uptake of optimal doses of IPTp-SP. The number of ANC visits made during pregnancy showed no important effect, suggesting that the quality of ANC counts more than just the quantity of visits. This means that the earlier a pregnant woman initiates ANC the more the time she has to be counseled on the dangers of MiP and the use of SP and other interventions such as mosquito bed nets [3,28]. This underscores the need to promote timely initiation of ANC among pregnant women in order to increase their chances for uptake of MiP interventions and subsequently avert malaria-related health outcomes. Beyond optimal use of SP for malaria control in pregnancy, timing of ANC initiations is associated with other important health outcomes [29-32]. As ANC remains largely a facility-based health care, the observation corroborates earlier findings about the imperative role of the health system in accelerating uptake of interventions, including SP against MiP [15], thus calling attention to strengthening the health system in such aspects as workforce sufficiency, skills, availability of equipments and supplies [15].

Between-district variations in the likelihood of partial and optimal use of IPTp-SP for malaria control in pregnancy were observed. Geographical factors, such as place of residence, have been acknowledged in other studies as important drivers or barriers to distribution, access and utilization of various health services [33,34]. While it is possible that factors that influence uptake of IPTp-SP may be inequitably distributed between the districts due to different contexts, it may as well be an indicator of how MiP interventions have penetrated the districts differently. It is also possible that some districts may be more rural or more urban than others as it is already documented that urban residence is associated with uptake of IPTp-SP. Therefore, programmes aimed at enhancing optimal use of IPTp-SP should fully account for area-specific contexts for better coverage and health outcomes.

Lastly, marital status was significantly associated with partial but not optimal use of IPTp-SP, whereby ever married women (currently divorced or widowed) were less likely than currently married women to have taken partial IPTp-SP doses. It is possible that married women have the advantage of discussing, planning and making decisions on their health matters with their spouses, which can encourage uptake of IPTp-SP. Some studies have shown a positive association between presence of inter-spousal discussion about health matters, such as family planning and utilization of various health services [35-37]. Therefore, women who are not married may have limited opportunity for discussions and decisions on health issues. However, further investigation is needed to find out the underlying mechanisms of this relationship.

Counseling on dangers of MiP and district of residence affected both partial and optimal use of IPTp-SP. While education affected only optimal use of IPTp-SP, marital status did so only on partial uptake of IPTp-SP. The effect of timing of ANC initiation was more pronounced for optimal IPTp-SP. Both occupation and maternal age had no significant effect on both partial and optimal IPTp-SP.

### Limitations

Some variables such as gravidity or fertility, rural/urban residence, religion, and socio-economic status of the respondents were not available for inclusion. Also information or recall bias may have happened because the data were based on interview only, as well as the two years delay within which the respondents may have had forgotten some details. No causal inferences may be drawn from these findings because of snapshot nature of cross-sectional studies.

## Conclusion

Optimal coverage of IPTp-SP of 44% in the study area could possibly increase to as high as 72% if sustainability was guaranteed for women who took only one dose. Improving health services especially ANC to ensure better counseling services on the dangers of MiP to pregnant women, proper timing of ANC initiation, formal education beyond primary school are necessary for enhanced coverage of IPTp-SP and consequently better health outcomes of pregnant women and their newborns in the study area.

More research studies especially those which are qualitative in nature are needed to elucidate any underlying social perceptions, beliefs, and prevention or treatment means of MiP. It is also important to understand why women who take only one dose of IPTp-SP do not take the second as recommended. This will divulge reasons for low coverage of optimal IPTp-SP. There might be some misconceptions concerning MiP interventions which may result in changing patterns of health care seeking modalities during pregnancy and consequently affect uptake of SP for IPTp.

## Competing interests

The authors declare that they have no competing interests.

## Authors’ contributions

AE conceptualized the problem, designed the study, performed data analysis and drafted the manuscript. GM, the Empower II Project leader, was involved in designing the study, solely sorted clinical issues around the problem, and reviewed the manuscript critically. SM, AM, IPK, and HK reviewed the manuscript critically. All authors read and approved the final draft of the manuscript.
